# The Music Playlist as a Method of Education Research

**DOI:** 10.1007/s42438-022-00319-y

**Published:** 2022-06-15

**Authors:** James Lamb

**Affiliations:** 1 University of Edinburgh, Edinburgh, UK; 2 Moray House School of Education and Sport, Holyrood Road, Edinburgh, EH8 8AQ UK

**Keywords:** Music, Playlists, Sound, Education, Methods, Research, Postdigital

## Abstract

As technologies are woven deep into the fabric of our postdigital society and universities, there is a need to devise new research methods, and to seek out new kinds of research material, in order to better understand our complex and changing surroundings. One such approach, I argue in this article, involves creating and analysing music playlists as a way of critically exploring the learning spaces and practices of higher education. To make this argument, I describe and discuss the ways that music playlists contributed towards an ethnographic study of undergraduate courses in Architecture and History at a UK university. This involved inviting students to participate in the creation of ‘study playlists’, as I sought to understand how their learning spaces and practices were affected by digital technologies. This approach initially helped to establish rapport and trust with participants, as well as eliciting conversation and interview discussion which surfaced how students use streamed playlists and other digital technologies to negotiate personalised learning spaces. By helping to reveal these and other rituals, the music playlist was shown to work as an ethnographic artefact, while at the same time exposing the postdigital character of the contemporary university.

## Introduction

I’ve got what I call a ‘music for work’ playlist and it’s got about 350 songs on it so far. So it's kind of just, hours! So I can shuffle it and know that the next song is always going to be something that I’m into. Or if I’m feeling like I want to like, focus in on something a little bit more, I know I have sections of the playlist that have this kind of music.

(Sandy McFall, Year 2 Architecture student)

For me, music is a nice way to get the motivation. It’s a nice accompaniment as well. Because sometimes it can get a bit monotonous when all you hear is the sound of your laptop, and the keys going. And if you’ve got music, you almost don’t feel like you’re doing work.

(John Brown, Year 2 History student)

In this article, I am going to propose that the music playlist, in concert with other qualitative methods, offers a way of productively exploring the learning spaces and practices of higher education. To make this argument, I will draw upon my Doctoral research, which was funded by the Economic and Social Research Council of the United Kingdom, and explored how the learning spaces of higher education are affected by digital technologies (Lamb 2019). Taking a qualitative, ethnographic approach, I spent an academic year observing the learning spaces and practices associated with second year undergraduate courses in Architecture and History within ‘Ancient City University’, a prestigious UK higher education institution. This included observing students and academic staff as they participated in a range of teaching and learning, and within a variety of settings across and beyond the campus. I sat alongside students in lectures and presentations, and as they participated in exhibitions, tutorials and workshops. They allowed me to observe their work in the design studio, library, computer lab, café and at home. These practices and environments were documented through audio field recordings, photographs and written notes, some of which were generated via a digital journaling method. This was followed by semi-structured interviews with five students and tutors from each course. Most relevant to the article presented here, though, was my use of student-generated music playlists.

This method helped to establish rapport and trust with students, something that I had previously found difficult to achieve. The playlists also provoked discussion before and during interview, thus working as a form of elicitation method. These conversations helped to expose the complex ways that a varied range of learning spaces were affected by technologies. This included the negotiation of personalised learning spaces in different corners of the campus, as well as the student’s ability to reconfigure ostensibly domestic, social and transitory settings into environments that were conducive to writing, reading, drawing and the performance of other educational activities. In this way, the playlist was shown to exist as an ethnographic artefact through the way it offered a way of interrogating the rituals, cultures and environments of learners.

## The Music Playlist

A ‘playlist’ in the context of this article refers to a sequence of music, mediated through a digital device such as a smartphone or laptop computer. The students who participated in my study almost exclusively accessed playlists via the Spotify streaming service, which advertises its ability to provide users with a vast range of music and audio content across a range of devices. On some occasions, students also listened to playlists hosted on the Mixcloud audio streaming service, or the Youtube video channel. My own interest in the critical possibilities of the playlist goes back to 2012 when I jointly established Elektronishes Lernen Muzik,^1^ a project where students, teachers and researchers can share their learning playlists, accompanied by explanatory ‘liner notes’ and representative ‘cover art’. The 25 contributions to date have offered fascinating insights into the ways that a wide variety of music is seen to productively accompany or inspire a range of learning activities. There is, though, a more straightforward way of witnessing the ritual of listening-while-learning, and the presence of the playlist within our learning spaces and practices.

If you happen to be on a university campus while reading this article, at the next convenient moment, take a few minutes to step away from your desk and stroll over to the library or a nearby computer lab. You could alternatively wander into a café or other social area (and if you are already in such a space, simply cast an eye around your surroundings). I am going to speculate that many of the students you encounter will be seen wearing earphones as they go about their work. It would be intrusive to interrupt their quest for knowledge; however, if you were to strike up a conversation with these students, I am confident that you would find a considerable proportion to be listening to a streamed playlist of music. This does not mean that every student favours musical accompaniment to their learning, but as I will come on to show, when the playlist is a commonplace feature within our educational surroundings, it certainly justifies our critical attention.

## ‘Spaces’ and ‘Practices’

In the pages that follow, the term ‘learning practices’ (used interchangeably with ‘learning activities’ to avoid repetition) is used to capture all the different activities I observed, or students in my study described, as they undertook their respective courses. In the case of Architecture students, this included, among other things, sketching designs on paper and screen, staging an exhibition, speaking about their work within a critical review and preparing a reflective portfolio. For History students, it included attending lectures, participating in tutorial discussion, doing background reading, writing essays and so on. This is not an exhaustive description of the activities that were performed in the two courses, and learning is obviously a great deal broader than what happens in and around the undergraduate classroom. The point, though, is that ‘learning practice’ is used in this article to describe any course-related activity undertaken by students.

The term ‘learning space’ is considerably more complex, particularly in the context of the postdigital university where technologies have blurred the distinction between formal and informal learning spaces (Boys 2011), and it is difficult and perhaps unhelpful to differentiate between educational activity that happens ‘on campus’ and ‘online’ (Nordquist and Laing 2015). In an earlier Special Issue of this journal concerned with the postdigital learning spaces of higher education, Lamb et al. (2022) noted that we can think productively about ‘learning space’ in terms of physical and online education environments, as well as the practices they support, and also in more philosophical ways. For the purpose of this article, though, ‘learning space’ is used to denote any setting where educational activity is performed. This includes the classroom and other conventional teaching spaces of the university, including the library, lecture theatre, design studio, computer lab and other spaces where students assemble. However, it also refers to the more domestic, social and transitory settings beyond the university’s physical estate, including the café, living room, train carriage and elsewhere. These are other learning spaces have been critically explored through a varied range of approaches which includes, but is not limited to, interview and conversation (e.g. Monty 2015), ethnographic observation (e.g. Johnson and Khoo 2018), journaling (Gourlay and Oliver 2016) and, as I will come on to discuss, through sound.

## Postdigital Thinking and Education

Alongside the working definitions provided above, I want to spend some time introducing postdigital thinking, as it may be an unfamiliar concept if you have arrived at this article primarily through an interest in methodology or music. Furthermore, while this is a methods article, the case for using music playlists in education research is made stronger by recognising how they are situated within the postdigital learning contexts of higher education. The concept of ‘postdigital’ can be traced back to two pieces of work that emerged simultaneously but independently towards the beginning of the century. Responding to what they saw as the ‘intellectual restrictions of the digital paradigm’ which resulted in ‘the reduction of continuous reality into discrete binary units’ (2000: 8), Pepperell and Punt proposed the ‘postdigital’ as a way of recognising that the digitisation of information and the presence of machines altered our daily lives, but not in a way that represented a clean break from the past. Around the same time, the scholar and composer Cascone described how a ‘post-digital aesthetic’ had emerged in response to working in environments suffused with digital technologies (2000), even if the term was already in use before his deployment of it in that way (Cascone and Jandrić 2021). Of particular interest to Cascone were the glitches and malfunctions of digital technologies that sometimes proved productive in the creation of new electronic music.

An important assumption in this formative work, and one that retains its currency as we critique and conceptualise contemporary higher education, is that counter to the popular notion of a ‘digital revolution’ where technologies are understood to have displaced previous materials and to dictate behaviour, postdigital thinking instead nurtures a more nuanced position, which sees emergent resources and approaches as having become ‘imbricated with our everyday actions and interactions’ (Feenberg 2019: 1). Thinking about education in particular, the concept of the postdigital thus provides a way of analysing and describing the relationship of the human learner (or teacher) to the technologies being collectively and individually experienced in the current moment (Jandrić et al. 2018).

The postdigital assumption that digital resources and practices have become a *regular* rather than remarkable feature of everyday life was neatly demonstrated as I spoke with Architecture and History students around their music-listening practices. Their matter-of-fact attitude to what I regarded as an incredible level of access to musical content can be seen as the product of their being born after the Internet boom of the 1990s, and the rapid ownership of mobile phones and personal computers that followed. Without generalising about their digital proficiency or preferences, unlike previous generations of undergraduates, these students had never reckoned with the finite number of cassettes that could be squeezed into a rucksack while packing for university. Their anytime-anywhere access to music, made possible by mass ownership of mobile devices, streaming services and an ability to seamlessly connect to the Internet, removed the dilemma of deciding which tape to slip into a Walkman ahead of an essay-writing stint in the library. Put simply, digital technologies and practices were woven into the fabric of their everyday experiences.

## Sonic Methods, Listening Space and Spotify: A Review of the Literature

The purpose of this literature review is to consider how music playlists are situated within the wider use of sonic methods and materials within education research. Consistent with the setting of this article, I have focused upon critical work that relates to music-listening and sound within higher education. Similarly, I am mostly interested in research that in some way helps us to consider the methodological possibilities of the playlist, rather than attempting to more broadly consider research around music in education.

## The Emergence of Sonic Methods

To date, sound has largely existed on the methodological fringes of qualitative and education research. This is a situation partly explained by what Gershon has described as the ‘ocular hegemony’ of inquiry (Daza and Gershon 2015: 640), and accentuated by ‘a lack of any real aural training in our culture’ (Chion 2012:53) which has swayed researchers to privilege sight over sound. There has been a tendency, Dicks et al. (2011) have noted, to regard sound as interview recordings that require transcription, or content to accompany images, rather than being more meaningful research material in its own right. Responding to this privileging of what researchers see over what they hear, Wargo et al. (2021) instead present a series of ‘sonic encounters’ where the conventional field recording, and the visually oriented nature of ethnographic representation and research itself, become reconfigured through a greater emphasis upon the aural. In common with Pink (2009). before them, Wargo et al. recognise the potential of using sound to convey, as well as to construct, knowledge. The possibilities of undertaking this kind of work have become considerably easier through recent advances in audio technologies (Dicks et al. 2011; Maeder 2013), not least on account of portable and relatively inexpensive hand-held devices that support the creation of high-quality recordings.

Turning attention to higher education research in particular, sonic material and methods are gradually becoming established as a way of critically tuning into our surroundings, a situation recognised by Gershon and Appelbaum (2018) who point towards the burgeoning body of work using sound to ask questions around contemporary educational activities, policies and theories. With a particular interest in the experiences, attitudes and practices of online learners, Bayne et al. (2013) invited a group of postgraduate students to capture audio of those spaces from where they connected with their institution. Recordings were submitted alongside photographs and explanatory text within ‘multimodal postcards’, and helped to surface a range of ways that these online students conceptualised their university, and sometimes fetishized physical buildings they would never visit. This was further explored with a specific emphasis on how these same learners used sound to construct space for learning away from the campus (Gallagher et al. 2016).

A different approach to exploring the relationship between learning, space and sound is provided by Wilson (2022) who proposes that we might use music theory to inform learning design and environments. With a specific interest in the hybrid learning that came to prominence amid the imposed constraints of the Covid-19 pandemic, Wilson suggests connections between the spatial and temporal representations of musical forms, and the organisation of components within learning design. Elsewhere, Ahern (2022) deploys the device of the ‘soundscape’ to consider the possibility of positively manipulating our online learning environments. Recognising the importance of the aural dimension of our educational surroundings, Ahern explores whether students and teachers might ‘plant’ sounds in those settings where online synchronous learning happens.

It is instructive to situate Ahern’s argument within the wider literature around higher education learning spaces. For at least the last decade, universities in the UK and elsewhere have, for a range of pedagogical and strategic reasons, invested vast sums of money redesigning their campuses (Goodyear et al. 2018; Mulcahy et al. 2015). However, while the library has been reinvented as a ‘learning commons’ (Johnson and Khoo 2018; Lomas and Olinger 2006) and the campus café has been reimagined as a place where students consume knowledge as well as coffee and snacks (Coulson et al. 2015), learning has at the same time been seeping through the boundaries of the university estate, supported by the emergence of new digital pedagogies and the proliferation of digital technologies across society. If we accept the need to create spaces that are conducive to learning, that digital technologies increasingly mean that these spaces exist beyond the campus and that learning spaces are in some way shaped by their sonic qualities (Neuman 2013; Gallagher et al. 2016; Ceraso 2018), there would seem to be a strong case for exploring whether and how we might try and nurture sound within and through our online learning environments. It is interesting to reflect on the possibility that educators and universities might seek to actively shape these learning spaces in the same way that music has been strategically used to influence behaviour in the shopping mall (Sterne 1997), factory floor (Bijsterveld 2012) and a multitude of other settings.

## Music for Learning

A further line of inquiry has been less concerned with how and where students listen to music, and instead whether it has an impact upon their comprehension. At least in the popular imagination, there has been enthusiasm for the possibility that listening to music might lead to some kind of improved academic performance or acquisition of knowledge. This has often been referred to as the ‘Mozart effect’, a term which emerged following a study by Rauscher et al. (1993) where, for a short period after listening to classical music, a group of college students were shown to have improved spatial reasoning. Among other approaches that have sought to establish whether a connection can be found between music and the performance of learning activities are de Groot’s (2006) study into the effects of background music on foreign language acquisition, Jäncke and Sandmann’s (2010) interest in the possible impact of music upon verbal learning, and Dolegui’s (2013) research into the impact of genre and volume upon cognitive performance. Elsewhere, Perham and Currie (2014) undertook a study that explored whether reading comprehension was improved when accompanied by the learner’s preferred style of music, and Bellier et al. (2019) more recently investigated the relationship between music and anxiety among medical students as they performed anatomical dissections. In light of the way that listening practices and learning preferences can vary across even a small group of students, it is unsurprising that these and related pieces of research do not present a clear or completely persuasive connection between music and the performance of academic tasks. What these and comparable studies do, however, is to reiterate the prominence of music-listening within our learning practices and spaces, such that it continues to be a cause for critical investigation.

## The Playlist and Spotify

As will become apparent below, the music playlist exists as a method but also the subject of research in itself. Situated within the field of music psychology, but also drawing on approaches from consumer psychology, Krause and North (2014) explored practices around music-listening devices and the strategies for selecting music. In particular, Krause and North were interested in whether psychological variables offered a better way of predicting music-listening practices than preceding approaches that had looked towards demographic and technological factors. Among other ways, this study is interesting for the way that it proposes technology, identity and behaviour to be connected, thereby striking a parallel with the postdigital assumption around the mutually defining nature of the biological, material, social and technological (Jandrić et al. 2018). Krause and North differently consider everyday listening practices through the concept of time (2016), which surfaced the possibility that playlist listening exists in the moment rather than something which is pre-planned and constructed. As I will come on to discuss, some students in my own research did indeed favour this kind of impromptu listening; however, others also meticulously compiled playlists to accompany their future learning activities.

Among the ten Architecture and History students with whom I worked most closely during my research, Spotify was the go-to music service for all but one of the participants, pointing towards its widespread presence within current listening practices. The emergence of Spotify has been accompanied by a range of critical work that has sought to understand its technical workings, the impact it is having upon the music industry and the kinds of listening practices it engenders. With an interest in exploring user experiences of curating, maintaining and consuming personal playlists, Hagen (2015) deployed a method that combined interviews, observation and the completion of self-reporting diaries. Among the findings to emerge from her study, Hagen noted that while Spotify and comparable services nurtured new ways of accessing music, playlist practices were also informed by pre-digital ways of collecting music. While some listeners in Hagen’s study were drawn to the immediacy and fluidity of the streamed playlist, others instead drew on more traditional music-collecting practices to seek out gems and curate collections that in some way represented their identity amid an abundance of listening content. Referring back to my earlier discussion around postdigital thinking, Hagen’s observation is interesting for the way that it presents emergent digital practices as coalescing with, rather than being distinct from, those that preceded them. Digital technologies, in this case in the form of the streamed playlist and the listening device, are shown to have reshaped rather than revolutionised practices around music-listening, thereby enacting a key aspect of Pepperell and Punt’s proposition of the postdigital (2000).

The relationship between existing and emergent music-listening practices was also a feature of Webster’s (2020) examination into the relationship between music streaming services and new forms of class distinction. Once again drawing on interviews with everyday Spotify users, Webster raises the question of whether streaming services and other online platforms extend or potentially destabilise class-based differences in how we interact with digital content and environments. Interesting from a postdigital perspective is Webster’s observation that counter to the ease of accessing digitally mediated music content, some users distinguished themselves by seeking out physical formats such as records. Therefore, digital practices and resources were shown to exist alongside pre-existing technologies and activities, rather than rendering them obsolete.

The social meaning of the playlist was considered in a different context by Boswall and Al Akash (2017), as they explored the role that revolutionary music played during a period of protest and turmoil. Working with a group of Syrian women living as refugees in Northern Jordan, Boswall and Al Akash studied the song choices on participants’ mobile phones, supported by interview conversation, to explore how music contributed to their everyday lives, their sense of self and their connections with the home country from where they had been displaced. This study is particularly helpful in supporting the case for the playlist as a research method through the way that collections of music are shown to act as a device for exploring wider activities and attitudes that include, but also extend beyond, listening in itself.

Rather than using music as a way of interrogating wider practices, Krukowski (2017) is among those who has raised questions about Spotify’s operating model, discussing for instance the royalties that find their way to the artists whose work can be found on its playlists. This question is further explored by Marshall (2015) who provides an overview of the controversy surrounding the payments made by streaming services to artists, but also makes the point that Spotify and other providers take an approach that aligns with wider practice among major record labels. As I touch on below, the popular use of Spotify to accompany student learning thereby contributes in a very subtle way to the commercialisation of those settings where educational activity is performed. Elsewhere, a range of studies have taken a more technical approach to interrogate how Spotify recommends content, although more salient from the perspective of qualitative educational research is Werner’s (2020) use of ethnographic work to explore whether and how Spotify’s algorithms have the effect of organising music along lines of genre and gender. As I will come on to discuss, the assumptions and interests that shape the algorithms beneath the Spotify interface potentially carrying implications for the ways the students negotiate personalised learning spaces, and the performance of activities that happen in these settings.

## Introducing the Music Playlist as a Method

When I first entered the Architecture design studio, I did so without a particular intention to investigate the listening practices of students. However, as I reviewed some of my early photographs, I was drawn to the recurring presence of music within student spaces, most often through headphones and earbuds, and occasionally through the sound of music itself.

Taking advantage of ethnography’s flexibility in enabling the researcher to pursue different paths of inquiry as they emerge, I added a form to my research website where these Architecture students could nominate pieces of music, alongside an explanation of how it contributed to their learning. I advertised this activity in conversation, as well as through a poster which I pinned to the ventilation column in the centre of the design studio. I presented this as a collaborative exercise, where I would compile and share a playlist of the nominated pieces of music.

An alternative approach was required in the case of the History students who did not enjoy a permanent space equivalent to the design studio where I could promote the activity, or be sure to find members of the group at any time of day, with the possibility of striking up a conversation around what they were listening to. Instead, I collected their listening preferences via email, screenshots of their Spotify playlists and during interview. Although my conversations with the group of History students about their listening practices generated rich insights, this feels less complete compared with the more involved approach I took with their counterparts in Architecture (and something which I address towards the end of this article).

**Fig. 1:**
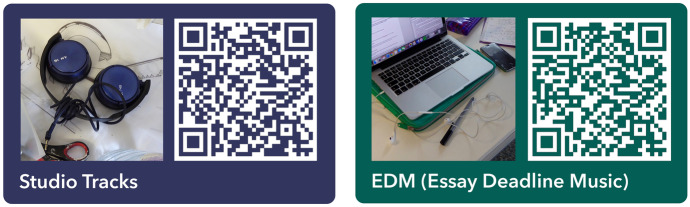
Linking to the playlists using QR codes

Across the two courses and these different approaches, fifteen students suggested pieces of music they listened to, sometimes offering an individual song, and on other occasions naming artists or genres or pointing me towards entire collections. From there, I created two playlists: ‘Studio Tracks’, made up of the songs suggested by Architecture students, and ‘EDM: Essay Deadline music’, in the case of History. I then published these playlists on the Mixcloud online streaming service, and subsequently made them available via QR code within my thesis (see Fig. 1).

## Rapport, Trust and Elicitation

An unexpected but welcome consequence of inviting students to participate in this activity was the establishing of rapport and trust within the Architecture group, something which I had previously found difficult to achieve, possibly on account of age difference, or our differing reasons for being in the design studio. Immediately after encouraging interaction around music, a number of students who had previously been cautiously cooperative became considerably more willing to engage in conversation, and enthusiastic about participating in other parts of my research. Without suggesting that playlists would provide common ground in every researcher-participant relationship, in these instances, music acted as a ‘hook’ that brought me closer to some of the students I was observing and working with. Although this was an unintended by-product of what I hoped to achieve through the use of playlists, I am keen not to underplay its significance, bearing in mind the importance attached to rapport and trust within successful ethnographic work (Cohen et al. 2011) and the way it can positively contribute towards what Hammersley and Atkinson recognise as the importance of managing ‘field relations’ (2007: 63).

The rapport and trust described here also contributed towards open conversation during interview, which included talking about playlists and music-listening more generally. Although it had not been my intention at the point of conception, the playlists emerged as a music-driven equivalent of the photo elicitation method, where images or other visual material are used to provoke reflection and conversation during interview. The photo-elicitation interview has become an established part of participatory ethnography according to Pink (2013), helped by the possibility of generating types of knowledge that might not be gained through observation (Hammersley and Atkinson 2007). In the case of my own study, participants were encouraged to explain their attitudes, experiences and practices around songs, rather than photographs, drawings or other forms of visual content. The degree to which this was a success is best evaluated through the insights that emerged from these conversations, two of which I will now move on to discuss.

## Reconfiguring the Classroom Through Sound

**Fig. 2:**
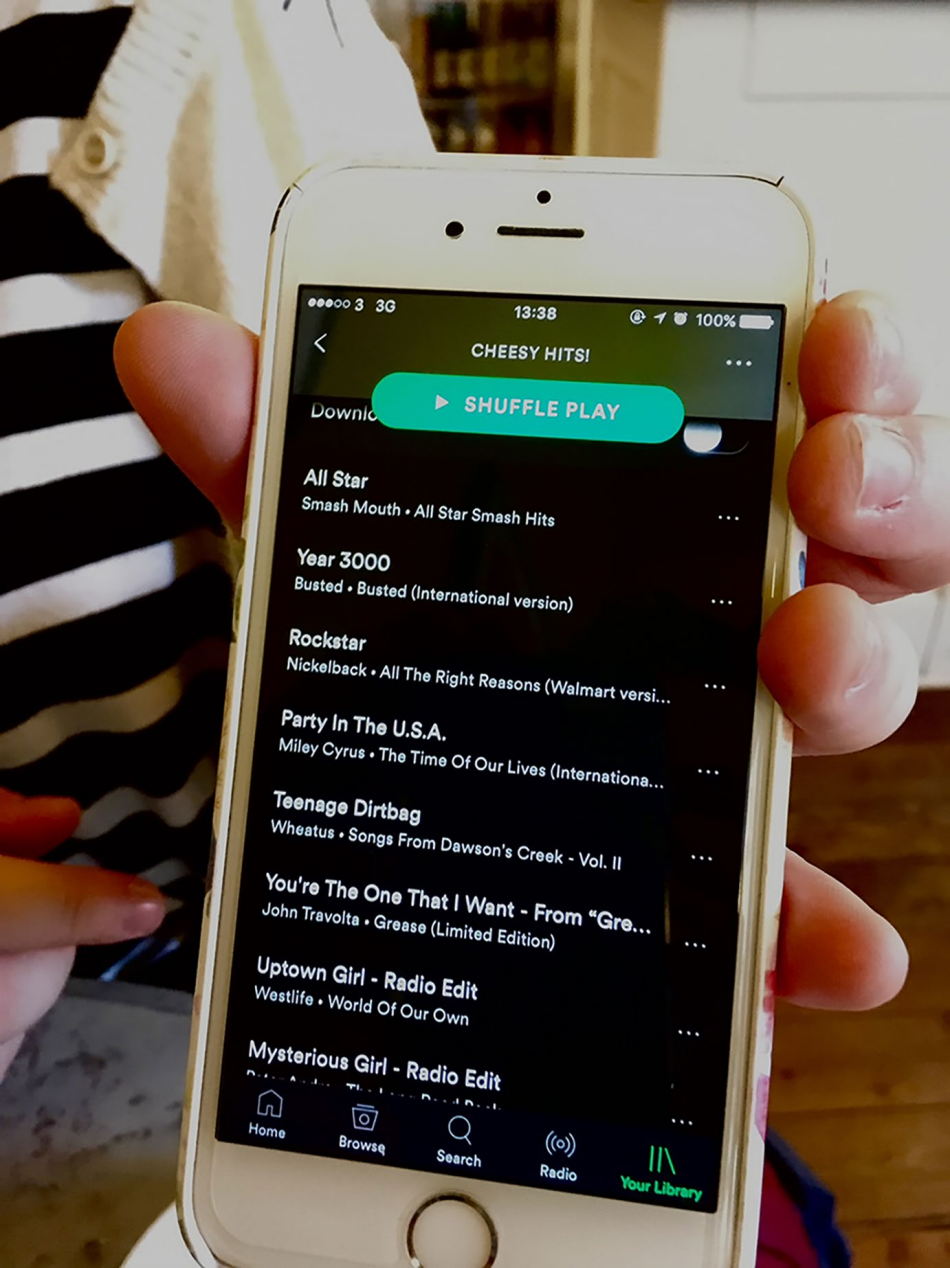
Heidi Green displays the Cheesy Hits! Spotify playlist that brought early momentum to her essay writing

One of the first steps in composing a History essay, Heidi Green told me, was to scroll through Spotify until she found the Cheesy Hits! playlist that brought some initial momentum to her writing. When the time came for refining her central arguments around the emergence of feminism in the United States, she would segue from Aqua and Ricky Martin into collections of movie soundtracks in order to allow for greater concentration. Over in the Architecture school meanwhile, Robbie Stanton preferred to accompany Computer-Aided-Design with dance music, as he sought to infuse his work with energy, and to enliven his surroundings. We get a sense here of the way that Heidi and Robbie sought to broadly synchronise the tempo (that is, the pace or beats-per-minute) of a song with the learning task in hand, something that was evidently straightforward through the vast amount of music available at the swipe of a screen. The temporal dynamic of listening-while-learning surfaced elsewhere in conversation as Sandy McFall described moving between playlists as afternoon merged into evening and he attempted to make progress on plans for his Architecture school. In contrast, with a History essay deadline fast approaching, John Brown favoured the up-tempo rock within a Mood Boosters Spotify playlist, in an attempt to defy fatigue and falling darkness (Fig. 2).

Like John Brown, other students described selecting playlists either to match or shape their mood, rather than necessarily trying to find the perfect accompaniment for a particular learning task. There is a psychological dimension to playlists here that surfaced elsewhere in conversation, with music being an enticement for students to sit down at the computer in the first place, to keep them there and to act as a kind of reward for a long stint at the keyboard. As I will come on to discuss, music was also seen as a way of aiding concentration, for instance by Ella Ness who turned to Spotify as a way of focusing on her reflective portfolio, while at the same time muting the conversation between friends in the adjacent area of the design studio that she might otherwise have been inclined to join. In the moments when Ella Ness, John Brown or any of their fellow students put in their ear buds and pressed play, we can see them establishing what Bull refers to as an ‘auditory bubble’ (2005: 344), where they used digitally mediated music to create a space that aligned with their learning needs in the moment.

Across all of these different motivations and approaches, the ability to find music suited to the particular learning activity was made possible by the vast amount of music available via the Spotify streaming service, and the simplicity and speed with which it could be accessed. This was neatly captured during conversation in the design studio, where Sandy McFall described using his Spotify premium account to continually extend a personal playlist that had grown to several hours-long, enabling him to dip into particular pockets of music to suit whatever the architectural task-in-hand might be. That said, the wealth of music could also have the opposite effect according to Sandy’s classmate Robbie Stanton, who explained that compiling the perfect playlist could become a form of procrastination, causing a slowing of progress on his plans for a public library.

Robbie Stanton’s assigned desk space meant that he had more reason than most of his peers to spend time seeking the right kind of music to soundtrack his learning. Situated adjacent to the corridor that ran the length of the design studio, Robbie’s permanent workspace was particularly subject to the aural intrusion of moving bodies and the continual hum of conversation, sounds that he and some fellow students described as ‘noise’, and Flügge refers to in her research around personal sound space (2011) as ‘sonic trespass’. The combined technologies of the streamed playlist, laptop computer and noise-cancelling headphones erected a wall of sound to supress what Robbie regarded as unwelcome elements of his physical surroundings. Even in the hushed atmosphere of the library, the shuffling of feet and paper was sufficiently distracting to Debbie Harris and Neville Smith that they would turn to playlists of ambient electronica and Edith Piaf as they sought to make progress on their History essays. Therefore, where the earlier examples suggest music being used to positively nurture an ambience for learning, in these instances, the playlist (in conjunction with connected technologies) was presented as erecting a barrier against sonic intrusion, and disruption from learning. The listening-and-learning strategies that students explained resonate with Ceraso’s argument that digitally mediated music-listening enables the individual to withdraw from unfavourable aural surrounding and to instead ‘customise their soundscape to match their moods and desires’ (2018: 87).

The experiences described here reveal insights into learner preferences and practices, as well as the temporal and spatial dynamics of the classroom. But they also hint at a form of power that the students exercised through digitally mediated music, or what Flügge (2011) describes as ‘spatial-acoustic self-determination’. Rather than accepting or adapting to the pre-existing ambience of the library or design studio, instead Debbie, Ella, Neville and their peers used sound to manipulate their assigned educational surroundings. When educators discuss the design of classrooms and comparable settings, conversation can understandably focus upon layout, sightlines and the material organisation of a particular physical setting. The approaches described by the participants in my research instead suggest a sonic rearrangement of space, where the furniture remains static and the student sedentary, but streamed music alters the ambience of the classroom and its propensity towards learning.

## Mobile Listening and Learning

Across the last two decades, the flow of data and mass ownership of digital technologies has meant that teaching and learning have increasingly been performed outside the established educational settings of the classroom and campus (Carvalho et al. 2016; Monty 2015). This has happened alongside the emergence of mobile learning as an area of research, with Sharples et al. (2009) and Pachler (2007) providing particularly useful introductions to this field. A central proposition of this work is that the portability of the modern computer device, combined with the growth of digital pedagogies and the widespread presence of educational material online, enables educational activity to be performed in a wider range of settings than was previously the case. Or to put it another way, the learner is less bound to the classroom and other designated educational spaces of the campus, as they are instead able to access content, and connect with colleagues and the university itself, through the flow of data and the mobile computing device. Within my own research, the conversations that took place around playlists surfaced music as a hitherto under-considered contributor towards the negotiation of learning spaces and the performance of learning practices in a range of ostensibly domestic, social and transitory environments. Settings as diverse as the café, train carriage, aeroplane cabin and couch of a shared student flat were consciously and temporarily reconfigured through music into spaces that supported writing, reading, note taking, drawing and other learning activities.

The potential for digitally streamed music to support a renegotiation of these settings echoes Bull’s work around mobile listening where he describes how the portable music device provides the user with ‘unprecedented power of control over their experience of time and space’ (2005: 343). Mobile listening, in Bull’s view, enables the nurturing of individualised narratives in a public environment, while Hosokawa (2012) similarly described how the Walkman offered a personalised sonic experience where the listener’s relations with their surroundings become altered. Although there is nothing new in students listening-while-learning, what is significant and different compared to the recent past is the ease of accessing a vast range of musical material. Therefore, when Debbie Harris passed time on a train journey by catching up on her History reading, and Matthew Redfearn developed his Architectural drawings while sitting at his grandfather’s dining table, they could scroll through Spotify until finding a playlist they felt matched the academic task in hand. As well as providing insights into some of the learning practices of these particular groups of students, these and other examples from my research were instructive in providing a wider understanding of the ways that space, technology and pedagogy coalesce.

When thinking about the flexibility and mobility that digital technologies have brought to learning spaces and practices, our thoughts might tend towards the possibility of remotely accessing educational content, or attending class when physically distant from the university. These are certainly valid questions to pursue; however, the examples presented here highlight how digital technologies provide possibilities beyond access and connection, to enable the establishing and rearranging of ostensibly domestic, social and transitory settings into productive, personalised learning spaces.

I have offered here two themes that emerged during conversations with students around music. Naturally, they shed a light on listening practices, but they also go further by providing insights into the temporal and spatial dynamics of learning. These themes also raise questions about the student’s power to renegotiate their surroundings, and the ways that digital technologies are woven through our everyday educational activities and surroundings. A fuller discussion of these ideas is presented within my thesis, alongside reflection upon other rituals and observations that emerged as students told me about their listening practices. For instance, although students evidently enjoyed the ability to sonically reconfigure their surroundings or retreat in an auditory bubble, they remained moored to their physical surroundings and its particular conditions and distractions. Meanwhile, student descriptions of how they selected and compiled playlists surfaced the influence of algorithms beneath the Spotify interface, which in turn raised the possibility that their learning spaces and practices were being shaped by profit and other interests that influence its design and code. Referring back to my discussion of the literature, I noted critiques of the commercial dynamics of the Spotify model, alongside work that has sought to understand how music is algorithmically selected and presented for consumption. On the basis that learning spaces and practices are in some way influenced by sound, this raises the intriguing question of whether the commercially motivated and algorithmically facilitated Spotify model is subtly shaping educational environments and practices through the interests of profit. When I pinned up my poster in the Architecture school inviting nominations for a course playlist, I did not anticipate that it would produce such a varied collection of insights into higher education learning spaces and practices.

## The Possibilities and Scope of the Playlist Method

In the previous section, I made the case that music playlists offer a way of critically exploring the learning spaces and practices of higher education. It is important, though, to also reflect upon the scope of the described approach, and to consider alternative ways that music playlists might contribute towards education research. As I have already noted, the absence of a permanent physical base for students on the History course meant that there were fewer obvious opportunities to invite students to contribute towards the creation of a playlist. Although this was a method-in-development, I recognise in hindsight that I could have used electronic means to promote the activity. Therefore, while conversation that took place around listening practices during interview with History students proved to be a rich source of insights, the approach feels less complete compared to that with the Architecture group, where working together on the creation of a collaborative playlist established rapport and trust, and provided more regular opportunities for open conversation.

## The Music Playlist in Concert with Other Methods

It also needs to be reiterated that I used music playlists as part of a wider ethnographic study. As I have explained, it was while reviewing photographs taken in the Architecture design studio that my attention was first drawn towards the presence of music. The meaning of these playlists and associated listening practices later became apparent during interview conversation. Therefore, while the use of playlists was of considerable value in establishing trust and provoking conversation, and contributed towards the emergence of insights around learning spaces and practices that might not otherwise have surfaced, this happened in conjunction with other established qualitative research methods.

In my discussion of the literature, I noted that sonic methods have been seen to exist on the margins of qualitative research, unable to vie with some of the prevailing approaches that are commonly used to interrogate learning spaces and practices. Perhaps, though, it is more productive to consider how sonic methods and practices can work *in concert*, rather than in competition with more conventional approaches to education research. Looking towards the research that provides the setting for this article, there will be moments where the music playlist can indeed take centre stage, performing a role that augments and goes beyond what can be achieved through observation, interviews and other more conventional forms of educational inquiry.

## Generalisability

As I have explained, my use of playlists was a response to the apparent prominence of music-listening practices among students participating in my ethnographic study. These were second year undergraduates, from two different courses, at a single UK university. I have been careful to avoid suggesting that the experiences of these research participants would be neatly replicated across other contexts. Although Architecture and History offer contrasting approaches to teaching and learning, other programmes and disciplines might require students to work in ways and in settings that do not lend themselves so readily to being accompanied by music. It is also important to recognise that these students experienced a high level of access to digital technologies that contributed towards the convenience of accessing music content: it should not be assumed that all learners would enjoy the same availability of laptop computers, smartphones and Internet coverage. It is certainly also the case that some learners see music as a form of ‘noise’ and thus something to be excluded from their writing or reading space, although this in itself is interesting from a research perspective. Therefore, the education researcher who has been left persuaded by the potential for the music playlist to support their own work might need to refine the method proposed here to synch with their own context and questions.

## The Playlist as an Ethnographic Artefact

If we accept that ethnography is broadly concerned with the study of cultures, rituals and people, then ‘ethnographic artefacts’ are objects that help us in this pursuit. Within a conventional educational setting, this might include the calculator and chalkboard, and the lab coat and laptop computer, among a multitude of other tangible materials to be found across and beyond the classroom. However, in the context of postdigital education where technologies are woven into the fabric of the university, there is a need to seek out the non-physical artefacts that are made possible through the flow of data: the email, the learning management system, the online journal article and so on. In light of the way that music playlists helped me to better understand the learning and listening practices of participants in my study, there is a persuasive case that they should be regarded as ethnographic artefacts.

## Analysis, Elicitation and the Wider Application of the Playlist Method

While my own study was concerned with learning spaces in particular, it is interesting to consider whether the playlist might work as a methodological device of value to researchers instead concerned with the influence of music upon learning *behaviour* (and I have noted this area of work in my review of the literature). For instance, approaching the production and perception of playlists from a psychological perspective might enable us to better understand those conditions (sonic and otherwise) that are seen to be conducive to writing, reading, reflection and other educational activities. At the same time, inviting conversation or participation in the creation of playlists might help to expose how music and other sounds are understood as ‘noise’ that disrupt concentration (as Gallagher et al. 2016, point towards). It is also intriguing to consider whether students might turn towards particular genres when performing tasks that require more pronounced physical activity (for instance when building models in the architecture workshop). In light of the postdigital assumption that the digital, material, biological and social are intrinsically connected (Jandrić et al. 2018), we might ask how the body, music and physical environment coalesce in the performance of particular learning activities. Among other applications, these kinds of knowledge could potentially inform the future work suggested by Ahern (2022) where sounds might be ‘planted’ in order to actively shape online learning spaces and support particular kinds of learning activities.

There would also seem to be a case for developing the music playlist as a form of elicitation method. Within my own research, the playlist contributed productively within a broader participant interview: the quality of insights that emerged from these discussions suggests that the playlist could potentially feature as the focal point of the interview and other discussions, something that Boswall and Al Akash’s (2017) study around listening practices among refugees points towards.

Finally, while the playlist method seemed to particularly synchronise with the interests of the undergraduates in my study, there is good cause to assume it could also be used to explore the attitudes, experiences and environments of other learners and educators. Taking the example of academic staff, as I have glanced at my Twitter feed in the course of writing this article, I have seen how educators are using playlists to collaboratively explore connections with nature and space,^2^ add a musical dimension to historical walking tours^3^ and provide a soundtrack to strike action that was taking place across the higher education sector.^4^ Therefore, among other lines of research inquiry, the playlist method advanced here might be used to explore conceptualisations of subject matter, augment our exploration of the urban environment and more generally capture the rituals and tensions of higher education within the particular moment.

## Conclusion

In this article, I have argued that researchers can use music playlists to interrogate the learning spaces and practices of higher education. To do this, I have reflected upon my own use of student-generated playlists as part of an ethnographic study that explored the relationship between digital technologies and learning spaces within undergraduate courses in Architecture and History. Inviting students to contribute towards the creation of playlists helped to build trust and rapport that I had previously found difficult to establish with some participants. The playlists also worked as a type of music-centred elicitation method, as they provoked conversation during and outside of interviews. This contributed to a range of insights around the ways that learning spaces and practices are affected by digital technologies, including how students negotiated personalised learning spaces across and beyond the campus. The playlist was shown to exist as an ethnographic artefact through the way it provided a method of interrogating attitudes, rituals and environments, and more generally the ways that technologies have become woven into the fabric of the postdigital university. Brought together, these observations demonstrate how the music playlist, in concert with other qualitative methods, can better enable us to critically tune into our educational surroundings.

## Footnotes

1 See https://www.elernenmuzik.net/. Accessed 10 June 2022.

2 See https://twitter.com/uhoutdoors/status/1470765850970013702. Accessed 10 June 2022.

3 See https://twitter.com/AcademicDiary/status/1498588929821396993. Accessed 10 June 2022.

4 See https://twitter.com/EmmakJackson/status/1499059045914886146. Accessed 10 June 2022.

## Declarations

The students who participated in my research gave their written consent to do. Pseudonyms have been used in the place of real names.
